# Supplementary material for: In Vivo Validation of Alternative FDXR Transcripts in Human Blood in Response to Ionizing Radiation
Source: Int J Mol Sci. 2020 Oct 23;21(21):7851. doi: 10.3390/ijms21217851 (PMC7660203; doi:10.3390/ijms21217851)
Supplement: Supplementary file 1 [file ijms-21-07851-s001.pdf]

Supplementary Material

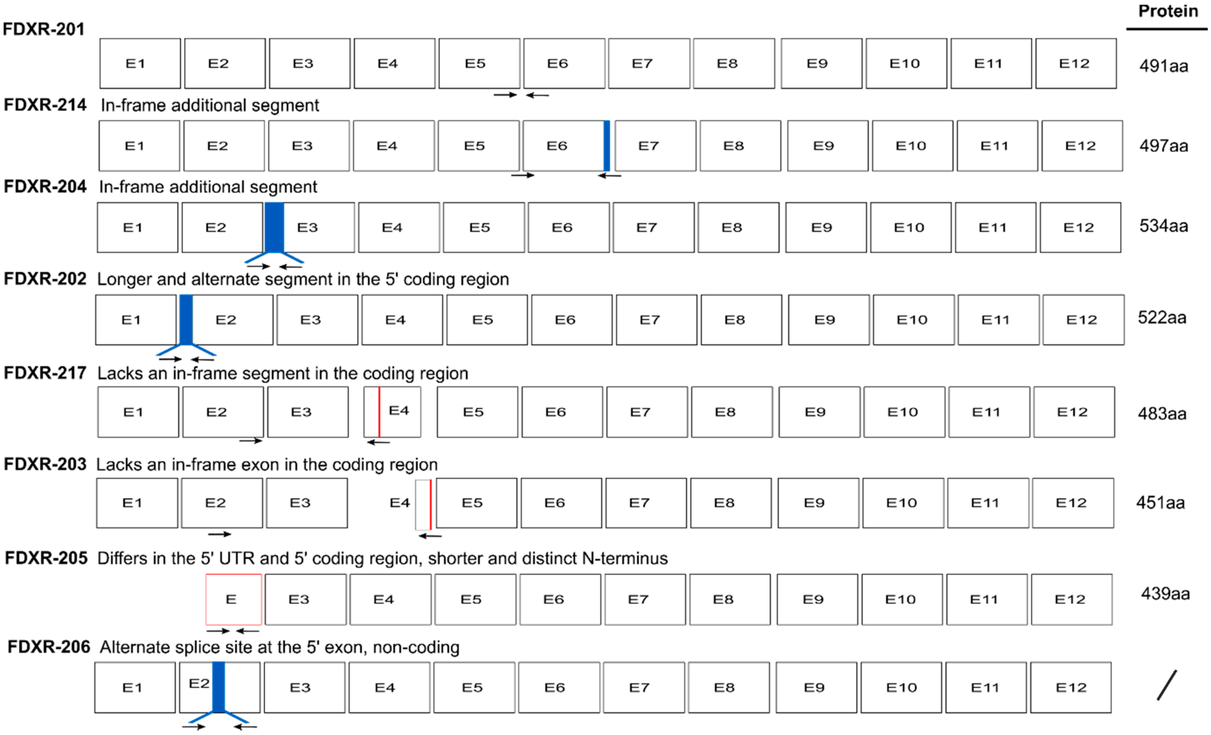

Figure S1. Position of the qPCR primers in the different FDXR variants. Primers sequences are described in supplementary Table 2.

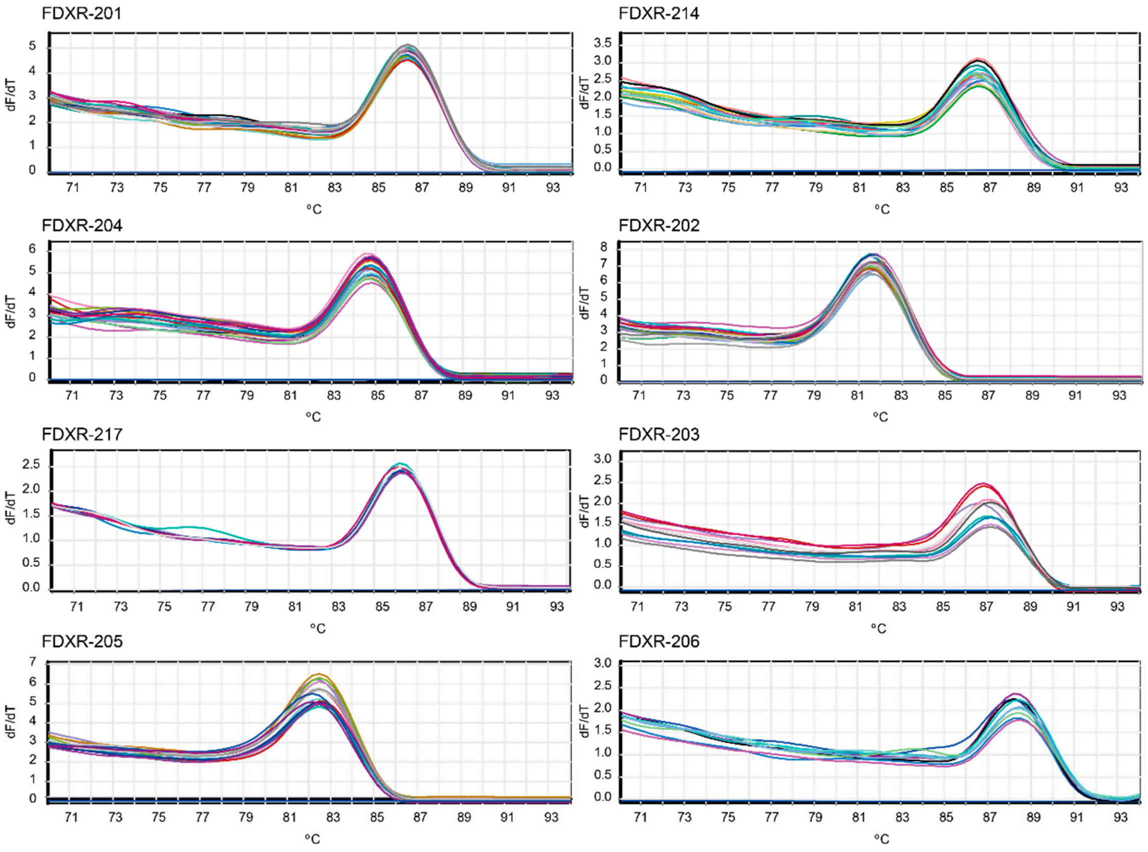

Figure S2. Melting curve analysis with SYBR Green-based qPCR analysis of 8 FDXR variants specific fragments amplified with specific primers (Table S2).

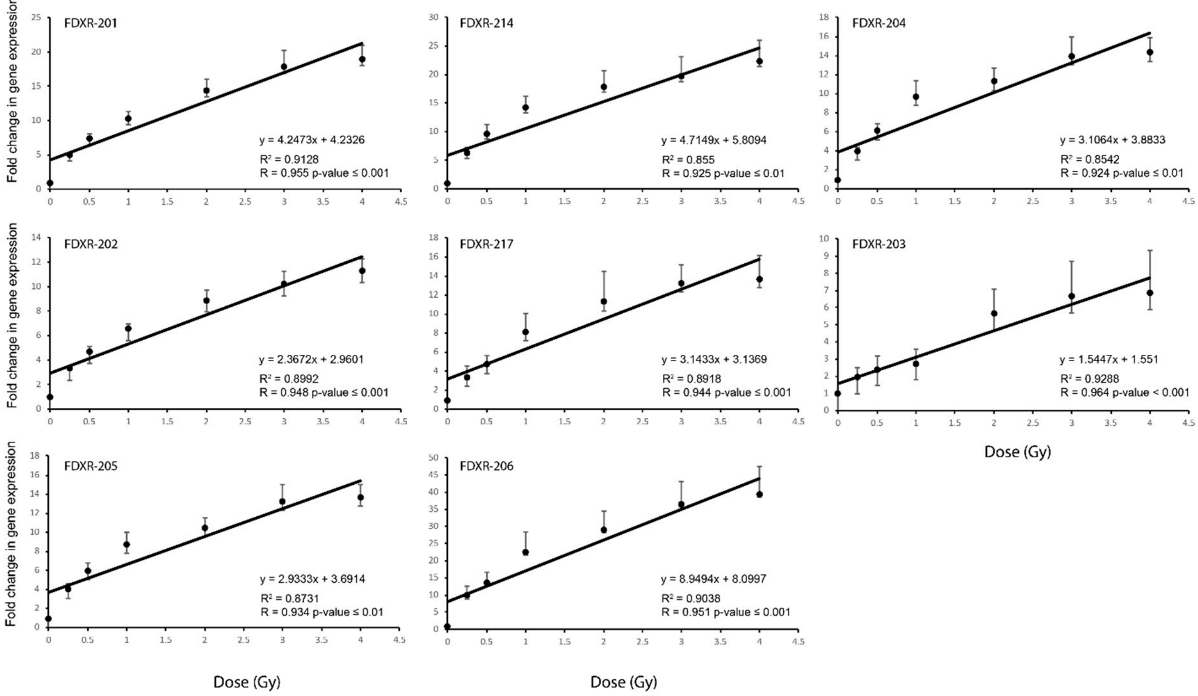

Figure S3. Linear regression analysis of the dose response curve for the FDXR and 7 variants for 10 healthy donors (5 women and 5 men). The data are presented as mean values ± standard error of the mean (SEM). Pearson's correlation coefficient and p-values are shown for each variant.

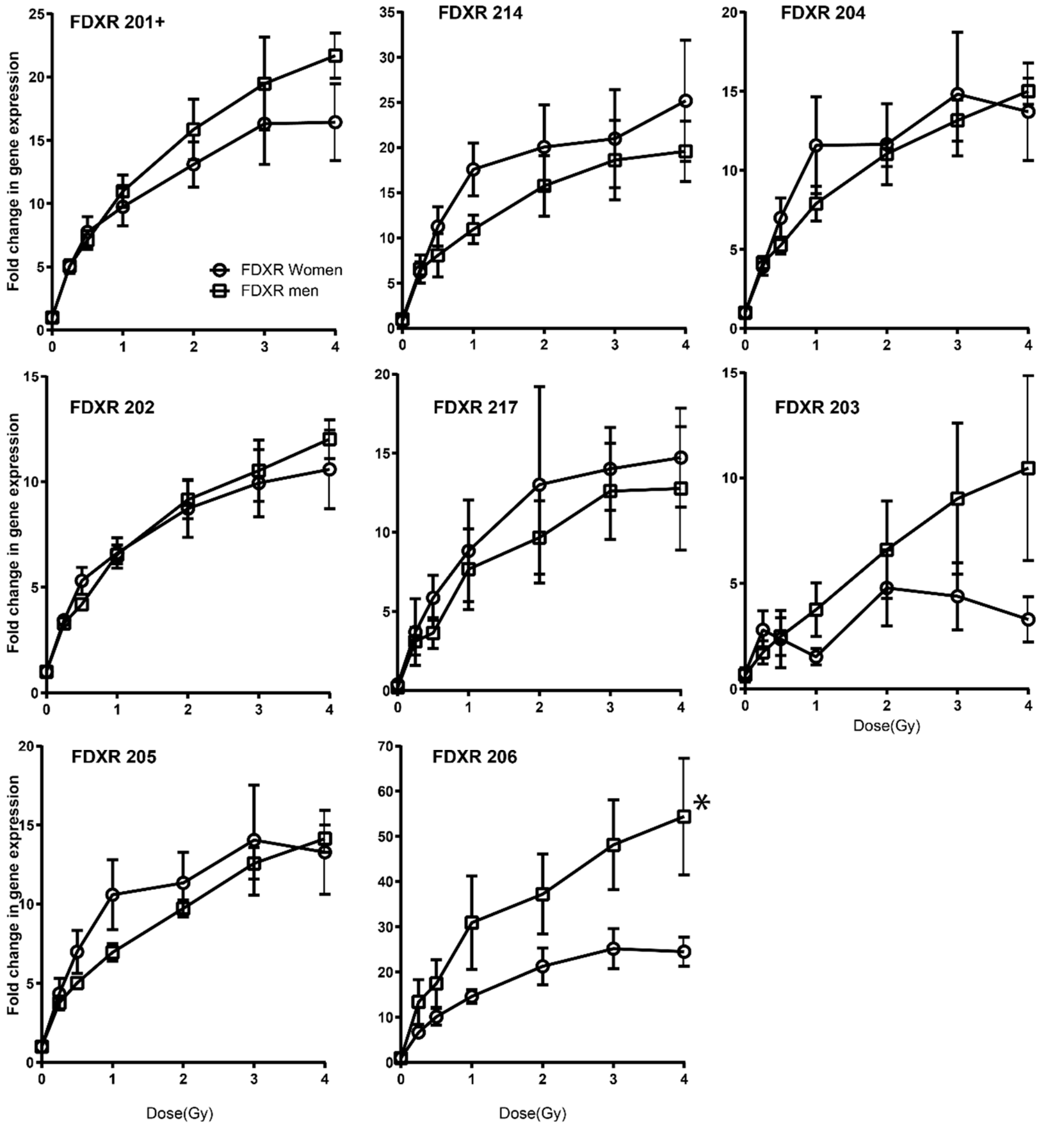

Figure S4. Gene expression of FDXR variants in human blood irradiated ex vivo in women and men. Blood from 5 women and 5 men was exposed to a range of x-ray doses (0.25, 0.5, 1, 2, 3, 4 Gy; dose rate 0.5 Gy/min) and incubated for 24h at 37°C. The data are presented as mean values ± standard error of the mean (SEM). Statistical analyses were performed in log-transformed data. Significant differences (T-test, $p \leq 0.05$) between women and men at each dose were indicated with an asterisk (*).

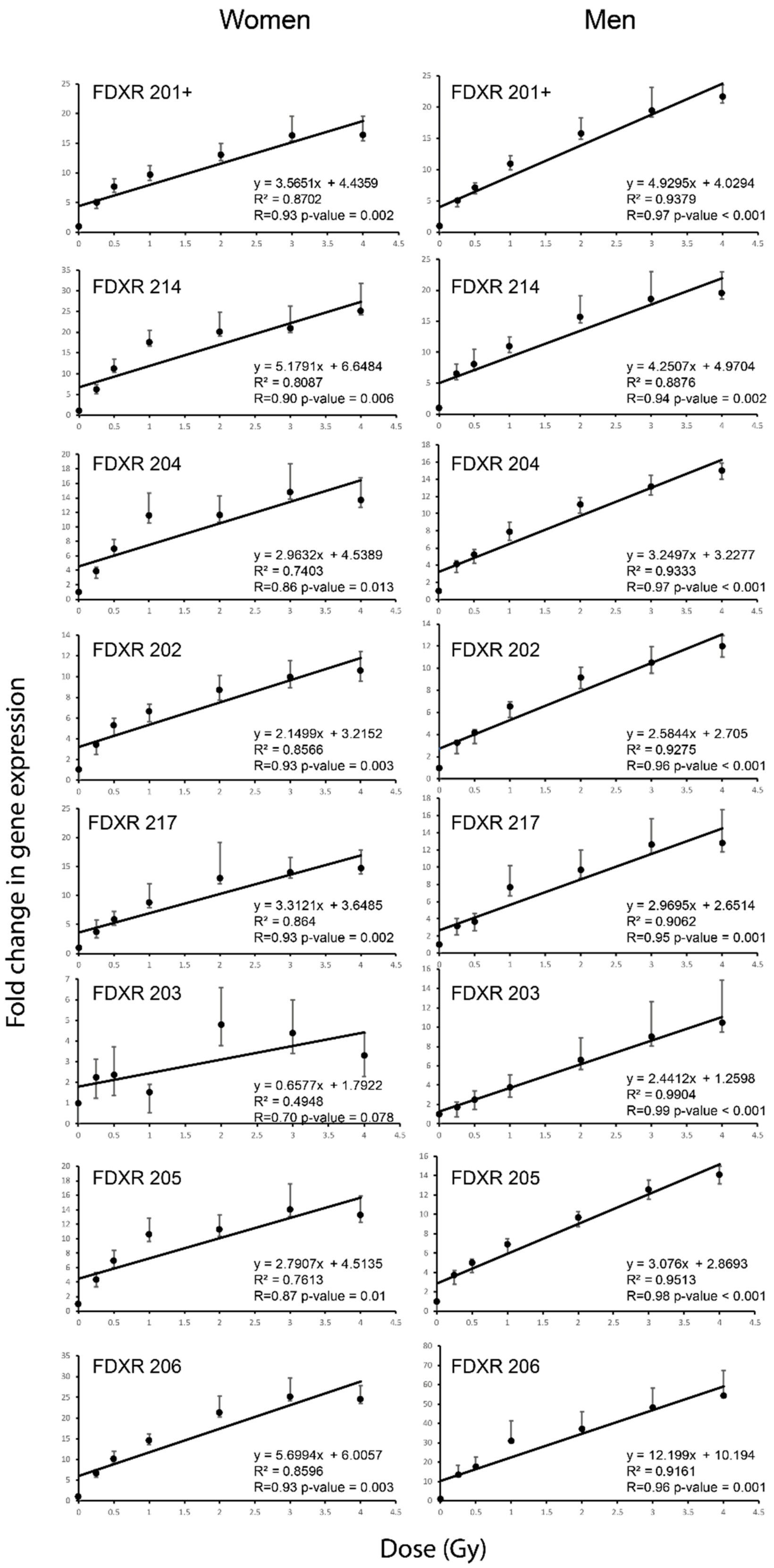

Figure S5. Linear regression analysis of the dose response curve for the FDXR and 7 variants for women (n=5) and men (n=5). The data are presented as mean values ± standard error of the mean (SEM). Pearson's correlation coefficient and p-values are shown for each variant.

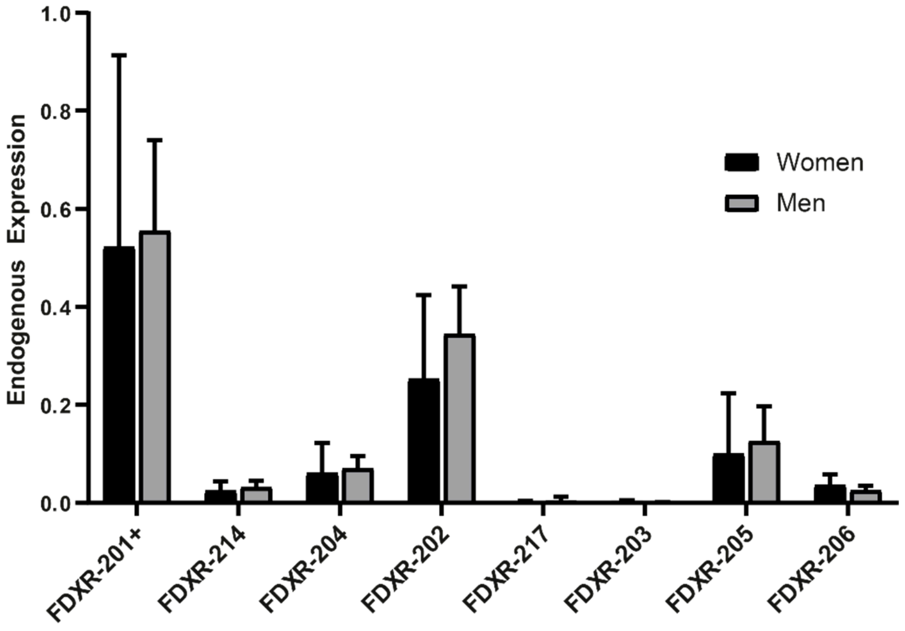

Figure S6: Comparison of basal level of expression of FDXR variants between genders. The basal level of expression of the FDXR variants was analysed in blood samples from 5 men and 5 women. The data are presented as mean values ± SD. Statistical analyses were performed in log-transformed data (T-test, $p \leq 0.05$). No differences were identified between genders.

Table 1. FDXR variants nomenclature, reference/transcripts ID, curation information, biotype and sequence and protein size.

Name Ensembl	GeneBank	NCBI Reference Sequence	Transcript ID	CCDS	Curated sequences	Biotype	bp	Protein
**FDXR-201**	**Transcript variant 1**	NM_024417.4	ENST00000293195.10	CCDS58593	✓	Protein coding	1846	491aa
**FDXR-214**	**Transcript variant 2**	NM_004110.6	ENST00000581530.5	CCDS11707	✓	Protein coding	1827	497aa
**FDXR-204**	**Transcript variant 3**	NM_001258012.1	ENST00000442102.6	CCDS58595	✓	Protein coding	1967	534aa
**FDXR-202**	**Transcript variant 4**	NM_001258013.3	ENST00000413947.6	CCDS58596	✓	Protein coding	1809	522aa
**FDXR-217**	**Transcript variant 5**	NM_001258014.3	ENST00000582944.5	CCDS58592	✓	Protein coding	1766	483aa
**FDXR-203**	**Transcript variant 6**	NM_001258015.2	ENST00000420580.6	CCDS58594	✓	Protein coding	1705	451aa
**FDXR-205**	**Transcript variant 7**	NM_001258016.3	ENST00000544854.5	CCDS58591	✓	Protein coding	1827	439aa
**FDXR-206**	**Transcript variant 8**	NR_047576.3	ENST00000577509.5		✓	Nonsense mediated decay	1912	46aa
**FDXR-207**			ENST00000577932.1			Retained intron	572	No protein
**FDXR-208**			ENST00000578473.5			Retained intron	2482	No protein
**FDXR-209**			ENST00000579482.5			Nonsense mediated decay	2157	46aa
**FDXR-210**			ENST00000579543.1			Nonsense mediated decay	558	46aa
**FDXR-211**			ENST00000579893.1			Protein coding	558	164aa
**FDXR-212**			ENST00000580492.5			Nonsense mediated decay	553	91aa
**FDXR-213**			ENST00000581219.1			Protein coding	567	178aa

**FDXR-215**	ENST0000058196 9.5	lncRNA	568	No protein
**FDXR-216**	ENST0000058271 0.5	Nonsense mediated decay	846	46aa
**FDXR-218**	ENST0000058388 1.5	Nonsense mediated decay	156 1	76aa
**FDXR-219**	ENST0000058391 7.5	Protein coding	176 2	463aa
**FDXR-220**	ENST0000061094 6.1	lncRNA	529	No protein

Table 2. Primers designs for qPCR analysis.

Transcript Variants for FDXR	Accession number NCBI	F Primer	R primer	Size (bp)	Cross-reaction with other FDXR variants
**FDXR-201**	NM_024417.4	5'-GTACAACGGGCTTCCTGAGA-3'	5'-CTCAGGTGGGGTCAGTAGGA-3'	121	FDXR-201,214,204,202,217,203, 205,206, 208,209,211,214,216,217, 218,219
**FDXR-214**	NM_004110.5	5'-CTGAGAACCAGGAGCTGGAG-3'	5'-GTCCGTTCTCTGGCACAAA-3'	143	
**FDXR-204**	NM_001258012.3	5'-GGTGGAAGCCTTGTGTTCT-3'	5'-GAGAGAGAGAGGCTGGGA-3'	94	FDXR-213
**FDXR-202**	NM_001258013.3	5'-TGAAGTAAGAGACCCTGCAAAT-3'	5'-ATATCCAACAGAAGCTGGAACT-3'	99	
**FDXR-217**	NM_001258014.3	5'-CTTCTACACGGCCCAACAC-3'	5'-AATGGGCCGTCTTCACCT-3'	131	FDXR-213/FDXR-218

**FDXR-203**	NM_001258015.2	5'-GCCACCATTTCTCCACACAG-3'	5'-CCCCGTAGCTCTTCACCTC-3'	198	
**FDXR-205**	NM_001258016.3	5'-AGGTCAGCCACGAGAGATAA-3'	5'-GCTCTCTGTCCTTATCTTCCATTC-3'	132	
**FDXR-206**	NR_047576.2	5'-CACCCCGAGTAGGAGCAG-3'	5'-GGAGAAATGGTGGCAGAAGC-3'	125	FDXR-208/FDXR209

Table 3. Tukey pairwise comparisons for Figure 1A. Grouping information for each radiation dose using the Tukey method and 95% confidence are presented in the table. On the left column all the variants are listed and in the top horizontal row the different irradiation doses groups are presented as in Figure 1A.

	**0.25 Gy**	**0.5 Gy**	**1 Gy**	**2 Gy**	**3 Gy**	**4 Gy**
**201+**	b	b	bc	bc	b	bc
**214**	ab	ab	ab	ab	b	b
**204**	b	b	bc	bc	b	bc
**202**	b	b	bc	bc	b	bc
**217**	b	b	bc	bc	b	bc
**203**	b	b	c	c	b	c
**205**	b	b	bc	bc	b	bc
**206**	a	a	ab	ab	a	a

Table 4. Tukey pairwise comparisons for Figure 2A (A) and Figure 2B (B). Grouping information for each radiation dose using the Tukey method and 95% confidence are presented in the table. On the left column all the variants are listed and in the top horizontal row the different irradiation doses groups are presented as in Figure 1A.

**A**	**0.25 Gy**	**0.5 Gy**	**1 Gy**	**2 Gy**	**3 Gy**	**4 Gy**
**201+**	a	ab	abc	ab	abc	ab
**214**	a	a	a	a	ab	a
**204**	a	ab	abc	ab	abc	ab
**202**	a	ab	bc	ab	bc	ab
**217**	a	ab	abc	ab	bc	ab
**203**	a	b	c	b	c	b
**205**	a	ab	abc	ab	bc	ab
**206**	a	a	ab	a	a	a

B	0.25 Gy	0.5 Gy	1 Gy	2 Gy	3 Gy	4 Gy
**201+**	b	b	b	b	b	b
**214**	ab	ab	b	b	b	b
**204**	b	b	b	b	b	b
**202**	b	b	b	b	b	b
**217**	b	b	b	b	b	b
**203**	b	b	b	b	b	b
**205**	b	b	b	b	b	b
**206**	a	a	a	a	a	a

Table 5. Average fold of change (FC), standard deviation (SD) and coefficient of variation (CV) of FDXR variants expression in response to radiation exposure in in vivo samples from total body irradiated patients (TBI). Total body irradiated patient's samples were collected 16 h after the first fraction and 8 h after the second (n=4). Gene expression profile of FDXR variants is presented in Figure 3.

	**TBI cancer patients**		
	**Average FC**	**SD**	**CV**
**FDXR-201**	5.8	3.5	100.6
**FDXR-214**	5.4	2.4	100.4
**FDXR-204**	10.1	12.0	101.2
**FDXR-202**	7.3	7.1	101.0
**FDXR-217**	5.9	4.9	100.8
**FDXR-203**	2.0	0.4	100.2
**FDXR-205**	7.8	8.5	101.1
**FDXR-206**	6.3	4.0	100.6

Table 6. Average fold of change (FC), standard deviation (SD) and coefficient of variation (CV) of FDXR variants expression in response to radiation exposure in in vivo samples from endometrial cancer patients treated with radiotherapy. Blood samples were collected 24 h after the first fraction (n=8). Gene expression profile of FDXR variants is presented in Figure 4.

	**Endometrial cancer patients**		
	**Average FC**	**SD**	**CV**
**FDXR-201**	3.1	0.6	20.3
**FDXR-214**	2.4	1.1	46.3

**FDXR-204**	2.9	0.8	28.1
**FDXR-202**	2.6	0.7	25.6
**FDXR-217**	2.6	1.2	46.3
**FDXR-203**	2.6	1.4	52.3
**FDXR-205**	2.6	0.5	19.6
**FDXR-206**	3.1	1.1	35.6

Table 7. FDXR variants identified by nanopore sequencing. Counts are normalized by HPRT1 and the coefficient of variation (CV) for their consistency between replicates has been calculated in the control and irradiated samples.

	**C1**	**C2**	**C3**	**Average**	**SD**	**CV**	**IR1**	**IR2**	**IR3**	**Average**	**SD**	**CV**
**FDXR-201**	0.03027	0.048509	0.032526	0.0371	0.0099	26.80	0.822521	0.715406	0.596453	0.7115	0.1131	15.89
**FDXR-214**	0	0	0	0.0000	0.0000	/	0.008203	0	0	0.0027	0.0047	173.21
**FDXR-204**	0	0	0.011765	0.0039	0.0068	173.21	0.09918	0.127171	0.102076	0.1095	0.0154	14.06
**FDXR-203**	0	0	0	0.0000	0.0000	/	0	0.003361	0.002163	0.0018	0.0017	92.51
**FDXR-205**	0	0.002021	0	0.0007	0.0012	173.21	0.008203	0.02577	0.004325	0.0128	0.0114	89.52
**FDXR-206**	0.001081	0	0.002076	0.0011	0.0010	98.67	0.01566	0.019048	0.028547	0.0211	0.0067	31.68
**FDXR-207**	0	0.001011	0	0.0003	0.0006	173.21	0.001491	0.005042	0.003028	0.0032	0.0018	55.88
**FDXR-208**	0.005946	0.012633	0.006228	0.0083	0.0038	45.74	0.134974	0.182073	0.202855	0.1733	0.0348	20.07
**FDXR-209**	0.000541	0.001011	0	0.0005	0.0005	97.79	0.014169	0.010644	0.013841	0.0129	0.0019	15.11
**FDXR-213**	0.003243	0.011622	0.004152	0.0063	0.0046	72.53	0.108874	0.103641	0.084343	0.0990	0.0129	13.06
**FDXR-218**	0	0	0	0.0000	0.0000	/	0.006711	0.001681	0.00346	0.0040	0.0026	64.56
**FDXR-219**	0	0	0	0.0000	0.0000	/	0.001491	0.006723	0.002595	0.0036	0.0028	76.54
